# Global gene expression analysis of lenses from different mouse strains and in the *α3Cx46* knockout mouse

**Published:** 2010-01-27

**Authors:** Yajun Tang, Thomas E. Crowley, Nalin M. Kumar

**Affiliations:** Department of Ophthalmology and Visual Sciences, University of Illinois at Chicago, Chicago, IL, 60612

## Abstract

**Purpose:**

Disruption of the mouse gene encoding the gap junction subunit α3 connexin 46 (*α3Cx46*) results in the formation of lens cataracts that have a severity affected by the genetic background of the mouse strain. To identify the genes that influence the severity of the nuclear opacity, global gene expression was analyzed in lenses from the 129SvJae strain and compared to the C57BL/6J strain.

**Methods:**

Lens transcripts were subjected to cDNA microarray analysis. Results on selected genes were confirmed by real-time PCR.

**Results:**

Genes that were determined to be altered in expression levels as a result of strain differences could be clustered into three groups: energy metabolism, stress response, and cell growth.

**Conclusions:**

There were no observed changes in gene expression as a result of the lack of *α3Cx46* in the different mouse strains, suggesting that the pathways mediated by this connexin do not influence gene transcription in the lens. Analysis of the transcript changes due to strain differences provides new insights into potential genetic modifiers of cataractogenesis. More detailed experimentation will be needed to determine if these observed changes do indeed affect cataractogenesis.

## Introduction

The mammalian lens is made up of three cell types: cuboidal epithelial cells, differentiating fiber (DF) cells, and mature fiber (MF) cells. The epithelial cells form a layer covering the anterior surface, and the cells at the equator differentiate to generate fiber cells by elimination of their nuclei and other organelles and elongate by expressing unique sets of proteins, including the crystallins. There are 16 distinct crystallins, and their abundance in the cytoplasm of fiber cells is critical for lens function [1]. DF cells migrate inward to become the MF cells in the center or “nucleus” of the lens [2,3].

During development of the mouse lens, gap junctional intercellular communication (GJIC) exists at epithelial–epithelial, fiber–fiber, and epithelial–fiber cell interfaces. Gap junctions are formed between adjacent cells by the contact of connexon hemichannels in each plasma membrane. Each connexon can be a hetero- or homo-hexamer of connexin (Cx) subunits. At least three connexin isotypes are expressed in the mammalian lens: α1x43, α3Cx46, and α8Cx50. It is well established that the epithelial cells express α1Cx43 and that there is a switch to α3Cx46 and α8Cx50 expression upon differentiation into fiber cells [4,5].

Like many other late-life diseases, such as Alzheimer disease and cancers, cataracts are conformational diseases in which there are unfolded or misfolded proteins that can form aggregates. In mice, targeted disruption of either gene coding for α3Cx46 or α8Cx50 resulted in cataracts. Disruption of *α3Cx46* induces age-dependent nuclear cataracts similar to human senile cataracts [6,7], while disruption of *α8Cx50* results in microphthalmia and zonular pulverent nuclear cataracts [4,5].

Column chromatography and immunoblot assays of lens protein preparations from wild-type (WT) and *α3Cx46* knockout (KO) mice with the 129SvJae background indicated that the opacity in the KO mouse is caused by aggregates of crystallin proteins that were at least partially induced by proteolytic cleavage of γ-crystallin [7]. At one month of age, γ-crystallin was detected in the insoluble fraction of lens homogenates from *α3Cx46* KO mice. A portion of the γ-crystallin (20 kDa when intact) is cleaved by a lens protease at this age, generating 9- and 11-kDa peptides. Moreover, the degree of the nuclear opacity in the *α3Cx46* KO lens was observed to be influenced by the genetic background of the mouse strains [8]. While *α3Cx46* KO mice on the 129SvJae background had severe cataracts associated with γ-crystallin cleavage, *α3Cx46* KO mice on the C57BL/6J background had far milder cataracts with no detectable γ-crystallin cleavage.

Recent technological advancements have made analysis of complex mixtures of transcripts as well as proteins feasible by microarray and proteomic approaches, respectively. Several transcript profilings of mouse lenses have provided insights into the molecular pathways that are differentially regulated during lens development, cataractogenesis, and in lenses of KO mice [9-16]. Proteomic methods have been applied to characterization of the mouse lens proteome [1,17-19]. Recently, using a global proteomic analysis of the C57BL/6J, 129SvJae, and the *α3Cx46* KO strains, several potential genetic modifiers for cataractogenesis were identified [20]. As a complement to this study, a global analysis of total lens RNA from these strains was also done and is reported in the current study.

The goal of the present study was to determine if there were differences in transcript expression between the lenses of two mouse strains (129SvJae and C57BL/6J) as well as between *α3Cx46* KO and WT mice. The former comparison is likely to identify potential candidate genes that prevent (or promote) cataract formation, whereas the latter comparison may provide insights into the mechanism by which cataract formation occurs in the *α3Cx46* KO mice. Microarray and real-time PCR approaches were used to determine these potential changes.

## Methods

### RNA preparation

The mice used in this study have been previously described [7,8] and were sacrificed by carbon dioxide asphyxiation followed by cervical dislocation. All animal experiments were performed according to the ARVO Statement for the Use of Animals in Ophthalmic and Vision Research as well as the guidelines established by the Animal Care Committee of the University of Illinois at Chicago. Lenses from 10-day-old WT or *α3Cx46* KO mice [7] with either a 129SvJae or C57BL/6J background were dissected in RNase-free medium, transferred immediately into extraction reagent (TRIzol; Invitrogen-Gibco, Rockville, MD) on ice, and stored at −80 °C. Briefly, pools of 20-30 lenses were homogenized in Trizol solution to lyse cells and solubilize the RNA. Then 20% (vol/vol) chloroform was added to separate aqueous and organic phases, followed by added equal volume of 70% ethanol to the aqueous phase. Finally, RNA was further purified using an RNAeasy Mini Kit (Qiagen, Valencia, CA,) using the buffers provided in the kit. RNA was eluted from the spin columns by using RNAase-free water. Concentration and RNA quality were assessed via spectrophotometry and denaturing agarose gel electrophoresis. All isolated total RNA had a 260/280 nm ratio of more than 1.8. RNA integrity was verified by inspection of the 28S and 18S rRNA bands on denaturing formaldehyde agarose gels (1.2%) after staining with ethidium bromide and determining by densitometry that the ratio of the bands was about 2 (28S:18S) as expected from the RNA length [7].

### Gene microarray hybridization

RNA samples for microarray analysis were prepared and hybridized to Mu74Av2 gene chips (Affymetrix, Santa Clara, CA) containing over 12,000 murine gene sequences as per the manufacturer’s protocol [21]. Briefly, total RNA was used for reverse transcription synthesis of double-stranded cDNA with the Superscript Choice System (Invitrogen-Gibco, Carlsbad, CA). The purified, double-stranded cDNA was then used for in vitro transcription, incorporating biotin-labeled nucleotide triphosphates with an RNA transcript labeling kit (BioArray High Yield; Enzo Biochem, New York, NY). The resulting cRNA was purified, fragmented, mixed with manufacturer-supplied control polynucleotides (a biotin-labeled oligonucleotide and four control cRNA sequences), and hybridized to the chip. After hybridization, the chip was washed, stained with streptavidin-conjugated phycoerythrin dye (Molecular Probes, Eugene, OR), and scanned on a chip reader (Affymetrix).

### Microarray data analysis

Affymetrix Microarray Suite (MS) 5.0 was used for data analysis. The statistical algorithms in the MS 5.0 suite process the raw GeneChip probe set data to generate expression values (Signals and LogRatios), detection and change calls, and associated p values. The data were scaled to a mean intensity of 250. To identify gene expression patterns that were significantly different between any two given groups, the following criteria were used: (1) a fold change ≥1.8; (2) at least one of the arbitrary expression levels was greater than 50; and (3) a *t *statistic, p≤0.01. Microarray data have been deposited on the GEO website (access number GSE5645).

### Real-Time PCR

Out of over 12,000 DNA sequences present on the chip, a statistically significant signal was obtained for about 3,000 genes using RNA from 10-day-old mouse lenses. The microarray gene expression data were verified by real-time PCR for the group of selected genes that are listed (Table 1). Total RNA was isolated from a minimum of six lenses from 10-day-old mice, and 2 µg of total RNA was used for generation of cDNA by reverse transcription using a kit following the manufacturer’s protocol (cMaster RT; Eppendorf AG, Hamburg, Germany). Briefly, total RNA was DNAse treated with RNase-free DNAseI (Promega, Madison, WI), and reverse transcription was performedusing random primers and the cMaster reverse transcriptase. After incubation at 50 ^°^C for 60 min, the reaction was stopped by heating to 85^ °^C for 10 min. Real-time PCR was performed on the cDNA with the product detected by using SYBR Green I dye (Invitrogen-Gibco, Carlsbad, CA). A SmartCycler (Cepheid, Sunnyvale, California) was used for real-time PCR. Hot-start PCR was performed by using HotMaster Taq DNA polymerase (Eppendorf AG). Each reaction (25 µl) contained 5 µl of cDNA dilution, 1× SYBR Green I dye, 2.5 mM Mg^2+^, 0.2 mM deoxynucleotide triphosphates, 0.1µM of each primer, and 1.25U HotMaster Taq DNA polymerase. Duplicate reactions were prepared for each dilution along with a no-template negative control (water control). The cycling conditions consisted of 1 cycle at 95 °C for 100 s for denaturation, followed by 40 three-step cycles for amplification (each cycle consisted of 95 °C incubation for 20 s, an appropriate annealing temperature [based on the primer sequence] for 10 s, and product elongation at 70 °C incubation for 20 s). The annealing temperature for each primer pair was 2 ^°^C less than the calculated melting temperature, which was determined by use of the program, Oligo Calc. The melting curve cycle was generated after PCR amplification. Each sample was analyzed twice by real-time PCR, and fold changes were calculated by averaging the fold increase from three individual RNA samples prepared from pools of lenses of the same genotype and age.

**Table 1:** Primers used in real time PCR analysis.

**Gene**	**Type**	**Sequence**
*18S*	Forward	AATTGACGGAAGGGCACCAC
* *	Reverse	GTGCAGCCCCGGACATCTTAAG
*Alox15*	Forward	CCACCACCTAAAACCAAGGA
* *	Reverse	ATGGCCACGCTGTTTTCTAC
*Bckdhb*	Forward	ACTCTAGTTGCCTGGGGCAC
* *	Reverse	GGTGTCATATCCGCAAACTCGA
*Car2*	Forward	GCAAGAGGCCATGTCTGCTC
* *	Reverse	GGAAGCGTGCGGCCTTTGCT
*Bfsp2*	Forward	GCCAAGTCCAGAGTCTCCAG
* *	Reverse	TCTGTAGCTGGCTCTTGCAC
*Col6a3*	Forward	ACAGCCAGACCTGCATTAGC
* *	Reverse	GACTTCTCGGGACACTCTCG
*Crybb1*	Forward	GGGACACCTGGACCAGCAGT
* *	Reverse	CGCCAGGCTCCAGCAGATA
*Ctsl*	Forward	TGAACCCAACTGTAGCAGCA
* *	Reverse	TCACGACAGGATAGCTGGC
*Epb4.1l4a*	Forward	CAAGCAGAGGAGGAGGTCAC
* *	Reverse	GTGTCTGGATCTCCGGTTGT
*Gas5*	Forward	GTGGGATCTCACAGCCAGTT
* *	Reverse	AATGAACAAGCATGCAACCA
*Hsp25*	Forward	TCTCTCGGTGCTTCACCC
* *	Reverse	ATGGCTTCTACTTGGCTCCA
*Tom1*	Forward	CACAGATGTGGGGAACAGTG
* *	Reverse	GTCCTTCTCCTGGGTCTTCC
*Tsc2*	Forward	GATCCAACCCCACTGACATC
* *	Reverse	GCCTGTTTCATAGGTGGGTG

Assuming rRNA levels did not change, the samples were normalized to the 18S rRNA. The validity of this assumption is supported by the real-time PCR results being similar to the microarray chip data, the latter being normalized to several different housekeeping genes. The specificity of the reaction was monitored by determination of the product melting temperature. The absence of significant additional peaks indicates that essentially only one specific product was generated. Semiquantitative agarose gel analysis of real-time PCR products of some of the selected genes also confirmed the microarray and real-time PCR data.

## Results

### Interstrain variation in transcript expression

Global gene expression analysis using cDNA microarrays was performed on cDNA prepared from lens mRNA preparations isolated from two mouse strains to identify transcripts with altered expression. Lens gene expression was compared between 10-day-old lenses from both the129SvJae and C57BL/6J mouse strains with either a *α3Cx46* WT or *α3Cx46* KO genotype (Table 2). The latter comparison was included to address the possibility that disruption of gap junctions due to the absence of *α3Cx46* might affect transcription of some genes. One objective of this study was to determine which genes may be involved in initiation or prevention of cataractogenesis. Thus, lenses from 10-day old mice were used because there were no observable cataracts at this age; cataract is typically detected at about 11.5 days in the *α3Cx46* KO mouse. By using lenses before the cataract, complications of the opacity affecting gene expression were avoided. For some of the genes that showed significant interstrain variation in expression, the microarray results were verified using real-time PCR on the same four cDNA preparations (i.e., 129SvJae and C57BL/6J with or without the *α3Cx46* gene disruption) (Table 3 and Table 4). In addition, real-time PCR assays were performed for some genes or proteins known to be expressed in the lens, such as *Hsp25* [20], but not included on the Affymetrix chip for microarray analysis.

**Table 2:** Gene expression changes greater than 1.8 in the lenses of 10-day-old C57BL/6J mice relative to expression in 129SvJae, in both WT and *α3Cx46* KO mice.

**Gene**	**Encoded protein**	**GenBank accession**	**Ratio (C57/129)**	
			***α3Cx46* WT**	***α3Cx46* KO**
**Growth regulation**				
*Gas5*	growth arrest specific 5	AI849615	17.1	13.5
*Myd116*	protein phosphatase 1, regulatory subunit 15A	X51829	3.4*	2.5
*Alox15*	arachidonate 15-lipoxygenase	L34570	2.4	2.9
*erdr1*	erythroid differentiation regulator 1	AJ007909	2.5	3.6
*Tsc2*	tuberous sclerosis 2	U39818	2.3	1.9
*SASH1*	SAM and SH3 domain-containing 1	AI837786	2.3	2.5
*Eif2s2*	eukaryotic translation initiation factor 2	AW125491	−1.9	−1.9
*ZDHHC5*	zinc finger, DHHC domain-containing 5	AI853561	−3	−2†
*ZFP728*	zinc Finger protein 728	AI152353	2.5	3.2
*Dbi*	diazepam binding inhibitor	X61431	−2.5*	−2.1
**Intracellular Trafficking**				
*Trappc5*	trafficking protein particle complex 5	AW120965	5.3	3
*Arl10c*	ADP-ribosylation factor-like 10C	AA822412	5.9	1.6*
*Tbc1d15*	TBC1 domain family, member 15	AI19433	2.4	5.3
*Tom1*	target of myb1 homolog (chicken)	AJ006972	1.9	2†
Metabolism and catabolism				
*Mod1*	malic enzyme, supernatant	J02652	2.4	1.8
*Atp7a*	ATPase, Cu++ transporting, α polypeptide	U03434	2.4	1.2†
*HGSNAT*	Heparan-alpha-glucosaminide N-acetyltransferase	AW125274	1.9	2.5
*Uble1a*	ubiquitin-like 1 activating enzyme E1A	AB024303	−1.6*	−1.9
*Ctsl*	cathepsin L	X06086	−1.8	−1.9
*Car2*	carbonic anhydrase 2	M25944	−1.9	−1.8
*Gad1*	L-glutamate decarboxylase	M55253	−2.2	−2.4
*Bckdhb*	branched chain ketoacid dehydrogenase E1,beta polypeptide	L16992	−2.7	−3.9
**Cytoskeleton**				
*Epb4.1l4a*	erythrocyte protein band 4.1-like 4a	D28818	2.5	2.6
*Tpm4*	tropomyosin 4	AI835858	−3.7	−3
**Cell signaling**				
*Pkig*	protein kinase inhibitor-γ	U97170	2.1	1.4*
*Gnb1*	guanine nucleotide binding protein, beta 1	U29055	−1.9	−1.9
*F2r*	coagulation factor II (thrombin) receptor	AW123850	−2	−1.9
*Uhmk1*	U2AF homology motif (UHM) kinase 1	AI846236	−2.4	−1.5†
**Transcription factor**				
*Gatad2a*	GATA zinc finger domain containing 2A	AI840824	2.7	2.2
*Ctbp2*	C-terminal binding protein 2	AW120820	1.8†	1.9
**Miscellaneous**				
*Col6a3*	procollagen VI- α3	AF064749	5.5	3.9
*Rsad2*	viperin	AA204579	6.5	5.1
*Fv4*	Friend virus susceptibility 4	C78850	4.9	8.9
*Rbp1*	retinol binding protein 1	X60367	3.5	2.9
*Hfe*	hemochromatosis	Y12650	2.9	2.7
*Snhg6*	small nucleolar RNA host gene 6	AA874329	2.6	4.4
*Sf3b5*	splicing factor 3b, subunit 5	AW047746	1.9	1.4†
*Cdv3*	carnitine deficiency-associated gene	AI837005	−1.7	−1.8
*Kctd12*	potassium channel tetramerisation domain 2	AI842065	−2.1	−1.5†
*Xist*	inactive X specific transcripts	L04961	−3	1.4
*Hebp1*	heme binding protein 1	AB013095	−7.2	−6.7
**Unknown**				
*BC056474*	cDNA sequence BC056474	AI853136	3.6	2.3
*6720463E02Rik*	RIKEN cDNA 6720463E02 gene	AI836322	3.5†	2.4
*1810058I24Rik*	RIKEN cDNA 1810058I24 gene	AI194274	−2.5	−2.5
*2510049I19Rik*	RIKEN cDNA 2510049I19 gene	AW258842	−4.8	−6.7
*1810037I17Rik*	RIKEN cDNA 1810037I17 gene	AW047207	−7	−6.1

*p<0.05, †p>0.05, all other data p<0.01.

**Table 3:** Expression of genes in the lenses of 10-day-old C57BL/6J mice relative to expression in 129SvJae in both WT and *α3Cx46* KO mice. Genes assayed by both microarray and real-time PCR.

**Gene**	**Encoded protein**	**Ratio (C57/129) by Microarray**		**Ratio (C57/129) by Real-time PCR**	
		***α3Cx46* WT**	***α3Cx46* KO**	***α3Cx46* WT**	***α3Cx46* KO**
*Alox15*	arachidonate 15-lipoxygenase	2.4	2.9	2.9	3.6
*Bckdhb*	branched chain ketoacid dehydrogenase	−2.7	−3.9	−13	−8.5
*Car2*	carbonic anhydrase 2	−1.9	−1.8	−3.9	−2.4
*Col6a3*	procollagen VI-α3	5.5	3.9	2.3	2.2
*Ctsl*	cathepsin L	−1.8	−1.9	−2.1	−2.4
*Epb4.1l4a*	erythrocyte protein band 4.1-like 4a	2.5	2.6	1.6	1.4
*Gas5*	growth arrest specific 5	17.1	13.5	4.4	3.8
*Tom1*	target of myb1 homolog (chicken)	1.9	2	1.5	3.2
*Tsc2*	tuberous sclerosis 2	2.3	1.9	2.1	2.3

**Table 4:** Expression of genes in the lenses of 10-day-old C57BL/6J mice relative to expression in 129SvJae in both WT and *α3Cx46* KO mice. Genes only assayed by real-time PCR.

**Gene**	**Encoded protein**	**GenBank accession**	**Ratio (C57/129) by Real-time PCR**	
			***α3Cx46* WT**	***α3Cx46* KO**
*Bfsp2*	beaded filament structural protein 2	NM001002896	6.6	10.9
*Crybb1*	crystallin, beta B1	NM023695	1.3	1.2
*Hsp25*	heat shock protein 25	L07577	4.3	4.5

The data obtained (Table 2, Table 3, and Table 4) corroborate the findings of other researchers [22,23] that transcripts encoding the beaded filament structural protein 2 (BFSP2, also known as cytoskeletal protein 49 [CP49] or phakinin) are dramatically reduced in the lenses of 129SvJae mice relative to C57BL/6J mice. Real-time PCR analysis showed that 129SvJae mice carrying *α3Cx46* WT have a 6.6 fold reduced level while those carrying *α3Cx46* KO have a 10.9 fold reduction in transcript levels for *Cp49* when compared to the C57BL/6J strain (Table 3 and Table 4). It has been previously determined that in the 129SvJ strain the *Cp49* gene contains a premature stop codon [22,23]. Filensen, a partner of CP49 in beaded filaments was not significantly altered in expression at the RNA level. In addition, significant differences in the levels of expression of 45 other genes were observed (Table 2) from about 3,000 genes that were determined to be expressed in the lens. The functions of the proteins coded by these genes are quite diverse, including apoptosis, growth regulation and differentiation, cell–cell and cell–matrix adhesion, cytoskeleton, protein synthesis, metabolic regulation, heavy metal ion transport, intracellular and intercellular signaling, transcription regulation, and intracellular trafficking.

Comparison of expression changes between the C57BL/6J and 129SvJae strains indicated dramatic differences for genes encoding: (1) the growth arrest specific 5 (*Gas5*) transcript [24-26]; (2) branched-chain ketoacid dehydrogenase E1-β (*Bckdhb*), which has a role in amino acid catabolism [27]; (3) heme-binding protein 1 (*Hebp1*); (4) procollagen VI-α3 (C*ol6a3*); and (5) heat shock protein 25 (*Hspb1*; *Hsp25*) [28]. Interestingly, there were also differences in expression levels for proteins involved in oxidation, such as cytoplasmic NADP^+^-dependent malic enzyme (*Mod1*), which transfers hydride ions from mitochondrial NADH to cytoplasmic NADP^+^ [29]; Cu^2+^ transporting ATPase (*Atp7a*), which plays an essential role in maintaining the full activity of Cu; Zn-superoxide dismutase 3 (S*od3*) through transporting copper to SOD3 in the trans-Golgi network [30]; and hemochromatosis (*Hfe*) transcript, which is involved in oxidative damage in the colon and mammary tissue in HFE-null mutant mice [31]. Disruption of the *α3Cx46* gene affected the magnitude of these changes in expression but not in its direction.

### Effect of a targeted disruption of α3Cx46 is not transcriptional

In contrast to the 45 transcript expression level changes detected between the 129SvJae and C57BL/6J strains, there was only a single transcript level change, namely protamine 1, detected in the lens between the WT and the *α3Cx46* KO mice. There was a tenfold increase in transcripts for protamine 1 in the lenses of *α3Cx46* KO mice compared to wild-type mice. This was independent of the strain background. The change in protamine 1 transcripts levels is likely due to the construct used to knockout the *α3Cx46* gene, which contained a protamine polyadenylation sequence in the 3’-nontranslated region of the neomycin gene. The lack of genes influenced by the disruption of the *α3Cx46* gene suggests that it is unlikely that changes in transcriptional regulation are of significance in the generation of the cataract in this model.

### Validation of the gene expression data

To validate the microarray data, the expression profiles of a few select genes were analyzed by real-time PCR (Table 3). Genes having different expression levels (both high and low ratios between 129SvJae and C57BL/6J strains) were chosen. There was excellent agreement in the determination of the fold changes between the two techniques of real-time PCR and cDNA microarray (Table 3). For one gene (*Epb4.1l4a*), the real-time PCR data showed the same direction of change as the microarray data, although the fold change was less (1.5× versus 2.5×).

## Discussion

Global gene expression analysis provides an unbiased method to identify factors that may have new functions during specific physiologic or pathological processes, such as cataractogenesis. In previous studies an age-dependent cataract has been shown to occur in the *α3Cx46* KO mouse [7], and this is dependent on the genetic background of the mouse [8]. To study the mechanism by which this cataract is formed and its dependence on the mouse strain, the lens transcriptosome was analyzed by using a microarray approach for studying the genes involved. An Affymetrix chip containing 35,000 genes was used, and 3,000 of the genes were detected in lens cDNAs. The transcript analysis between the different mouse genetic strains indicated expression changes in genes that encode for chaperones, antioxidants, cell proliferation, protein synthesis and degradation. Based on this analysis, we propose a hypothesis that accounts for the prevention of cataractogenesis in the C57BL/6J strain. In this hypothesis, the production of reactive oxygen species (ROS) and the modulation of their harmful effects on cell physiology by antioxidants and chaperones are suggested to be the major differences between the lenses of the C57BL/6J and 129SvJae strains. We further speculate that HSP25 is responsible for reducing the severity of the cataract upon disruption of *α3Cx46* in a C57BL/6J background.

Because the cataract in the 129SvJae *α3Cx46* KO strain is observed in the nucleus of the most terminally differentiated region of the lens that lacks active RNA and protein biosynthesis, it is perhaps not surprising to find only a few genes are differentially expressed when transcripts from *α3Cx46* KO mice were compared with those from wild-type mouse lenses. This is consistent with the conclusion that the cataract phenotype is a result of posttranscriptional changes rather than changes in transcript expression caused by disruption of *α3Cx46*. Thus, our results indicate that disruption of the *α3Cx46* gene does not significantly effect expression of the genes analyzed in this study. This also suggests that changes in pathways caused by disruption of the *α3Cx46* gene do not influence gene transcription in the lens and that this is independent of the strain background.

The epithelial cells along the equator of the mammalian lens continue to proliferate throughout life [2], and the mass of the mouse lens continues to increase until at least 12 month of age [7]. As the epithelial cells exit this cycle and differentiate into fiber cells, elimination of the nucleus causes cessation of transcription, but protein synthesis increases to provide a high level of crystallin and other fiber cell-specific proteins [3]. As the DF cells migrate toward the nuclear region to become MF cells, endoplasmic reticulum, Golgi, and ribosomes are degraded, causing a shutdown of protein synthesis [32]. The high level of protein synthesis in the DF cells implies a high metabolic rate requiring energy, while the degradation of the organelles necessitates catabolic enzyme activity. The data presented in Table 2 indicate that the genes involved in the rate of protein synthesis, metabolism, catabolism, and epithelial cell proliferation are decreased in the lenses of 10-day-old C57BL/6J mice relative to 129SvJae mice.

For example, accumulation of the *Gas5* transcript is an indicator of reduced protein synthesis [24-26], while the protein coded by *Tsc2* functions as a protein synthesis inhibitor [33-35]. Similarly, SAM and SH3 domain-containing protein (SASH1) is suggested to have a tumor suppression function [36]. Further evidence for a lower rate of protein synthesis in the C57BL/6J mouse lenses is supported by the fact that transcripts for eukaryotic translation initiation factor 2-subunit b2 (*Eif2s2*), metabolic enzymes (such as carbonic anhydrase 2 [*Car2*]), and catabolic enzymes (such as *Bckdhb* as well as the lysosomal cysteine peptidase cathepsin L [*Cts1*]), were shown to be reduced in this strain relative to 129SvJae mice. The reduced protein synthesis may result in changes in cell proliferation and apoptosis. In combination, the results presented in the current study are in agreement with the observations in a previous study that concluded that the C57BL/6J lens is “developmentally retarded” [37].

Oxidation of key cellular components is likely to have a major role in age-related nuclear cataractogenesis [31]. Long-term preservation of lens clarity may depend on the maintenance of hypoxia in the lens nucleus. The signaling pathway that leads to crystallin aggregation may include ROS. Since the concentration of ROS in MF cells is proportional to the rate of the various metabolic processes, production of these reactive species may be greater in the 129SvJae lenses since this mouse strain has increased expression of genes involved in metabolism and catabolism when compared to the C57BL/6J strain.

In addition, several transcripts that encode for proteins that synthesize or have a role as antioxidants were determined to be altered in their expression levels in lenses of C57BL/6J mice relative to 129SvJae mice. These include transcripts for the proteins MOD1 [29], SOD3 [30], and HFE [38,39]. The observed increased expression of these genes in the C57BL/6J lens may prevent damage by ROS.

Our real-time PCR data demonstrate that expression of the *Hsp25* gene in the lenses of C57BL/6J mice is higher than in the 129SvJae strain. This has also been confirmed by proteomic methods [20]. The induction of Hsps is a well recognized feature in the cellular response to stress and pathological conditions.

HSP25 can also induce the production of glutathione in cultured mammalian cells, thereby providing protection from the harmful effects of ROS [40]. HSP27 (human analogue of HSP25 in mouse) is important in maintaining intracellular redox homeostasis by keeping glutathione in its reduced form and by decreasing iron intracellular levels [41].

The role of HSP25 in maintaining homeostasis in many cell types has been demonstrated [42,43]. We suggest that a similar role for HSP25 may be responsible for inhibiting cataractogenesis in normal lenses. It is likely that cataract formation is influenced by the redox capacities and protein aggregation induced by proteolysis within the lens. This is supported by the fact that heat shock factor 4 (*Hsf4*) KO mice develop cataracts during the early postnatal period and that *Hsp25* gene expression level is decreased several hundred fold in *Hsf4* KO mice [44,45]. The reduction in HSP25 may act as a modifier for lens opacity.

*Hsp25* can be regulated by several transcription factors, including HSF4, hypoxia-inducible factor-1 (HIF-1), signal transducer and activator of transcription 3 (STAT3) and activating transcription factor 5 (ATF5) [46-49]. Analysis of the microarray data in the present study indicate that the expression levels for *Stat3* and *Hif-1* transcripts were unchanged in the different mouse strains, whereas both *Hsf4b* and *Atf5* transcripts were increased by 1.5- and 1.7-fold, respectively, in the C57BL/6J lenses compared to the 129SvJae lenses (data not shown). This will need to be confirmed by real-time PCR.

As we previously demonstrated, a lens-specific isoform of calpain-3, Lp82, is important in the development of the cataract in the 129SvJae *α3Cx46* KO mouse [6,50]. Diazepam-binding inhibitor (Dbi) has been shown to be a component of the Ca^2+^-dependent proteolytic system, which promotes activation of m-calpain [51]. Our finding of a lower expression level of the *Dbi* gene in the 129SvJae strain suggests a model in which the Ca^2+^ ion concentration needed to activate Lp82 is lowered in this strain.

The initiating factor in cataractogenesis in the *α3Cx46* KO mouse is most likely the increased cytoplasmic Ca^2+ ^concentration in their MF cells. However, it is not known if this triggers proteolysis of γ-crystallin resulting in its aggregation or if the increased Ca^2+^ concentration causes proteolysis downstream of the aggregation. In either case, the greater abundance of HSP25 in the C57BL/6J lens may have a key role in preventing the initiation and/or severity of the cataract in this strain upon disruption of *α3Cx46*. A link between Ca^2+^ and HSP25 function has been proposed for contraction of esophageal smooth muscle [52], and a similar mechanism may occur in the lens. The increased Ca^2+^ in MF cells of the α3Cx46 KO mice may prevent the crystallin degradation and/or aggregation-inhibition function in the C57BL/6J strain because of the greater abundance of HSP25 in this strain. Alternatively, rising Ca^2+^ may induce the chaperone function of HSP25.

In summary, the lack of changes in the gene expression levels in the lenses between WT and *α3Cx46* KO mice suggest that there are no significant changes in transcriptional regulation in the lenses upon disruption of the *α3Cx46* gene in mice. However, analysis of the changes in the gene expression levels between the different background strains suggest that HSP25 and the predicted lower rates of protein synthesis, metabolism, and catabolism in the fiber cells of the C57BL/6J mouse protects this strain upon disruption of *α3Cx46 *from a more severe nuclear cataract when compared to the 129SvJae strain.
